# Original Translations

**Published:** 1883-06

**Authors:** 


### Translations from Foreign Exchanges.

BY O. STROINSKI, M.D.

THE EFFECTS OF RUSSIAN BATHS.

Scientific researches as to the influence upon the human system of Russian baths, have recently begun, and Tumas. Sasjetsky, Fjalkoffsky, Tarehanoff and others have published their experiments. Dr. Godlewski has recently made extended experiments on two healthy persons for twenty days, with and without the application of heated birch-rods. In all the experiments, the air was not entirely saturated with hot steam, and the examined persons were equally nourished, and had an amount of work to do sufficient to preserve the regular "nitrogen equilibrium." For five days before the first series of experiments, the two persons were thoroughly examined. Then a series of baths was given for ten days without application of birch-rods, after which followed a series of baths with birch-rods for ten other days. After this time, the persons were closely observed for five days. The experiments were made in the following order. The patients having arrived in the bathing institute, 1, the pulse was counted exactly over the radial artery; 2, the number of respirations was determined; 3, they were weighed; 4, the capacity of the lungs was measured with the spirometer (three times); after having undressed, they rested for a few minutes, and then, 5, the temperature in the axilla and the rectum was taken; 6, the force of inspiration and expiration was measured by the masked pneumatometer, the highest numbers being noted; 7, the circumference of the chest was measured with a leathern strip on places which had been marked with nitrate of silver; in front, on the fifth rib, and on the back at the superior angle of the scapula, the arms being horizontally raised, (a) in regular breathing; (b) in deep inspiration; (c) in total expiration; 8, with the same strip was measured the circumference of the abdomen and of the upper and lower extremities; 9, the muscular force was determined, (a) in the hands by pressing upon a hand-dynamometer; (b) in the feet by pressure upon an oval dynamometer which was fastened to a board; (c) the force of the body was measured by connecting the latter dynamometer with a hook-formed mechanism, the hands pulling the hook. The two persons were then sent to the bathing room to be soaped (middle temperature of the first series being 38.6° Celsius, and of the second series 44.4° Celsius). They washed themselves with warm water (first series 47.95, and second 45.10 Celsius). Twenty minutes after being soaped, the pulse and respiration were counted, and the temperature in the axilla and the rectum was measured. Then they stepped upon the elevated bank; in the first series but lying on the bank; in the second series being whipped with the birch-rods (temp. 64.1° C.). After this repeated pneumatometric and dynamometric measures were taken. The urine was collected every 24 hours, the specific gravity determined with the urinometer, as also the quantity of nitrogen, phosphoric and sulphuric acids present. The results of these very minutely executed experiments are as follows: 1. The pulse was accelerated. 2. The respiration increased. 3. The circumference and the expansion of the thorax enlarged. 4. The force of inspiration and expiration were diminished. 5. The capacity of the lungs was lessened. 6. The temperature in the axilla and the rectum was higher. 7. The weight of the body was considerably diminished. 8. The circumference of the arms and legs was enlarged. 9. The circumference of the abdomen was diminished. 10. The muscular force in the hands was not very much decreased, but that of the legs and the trunk considerably. 11. The quantity of urine in 24 hours was diminished, but the specific gravity increased. 12. The quantity of nitrogen was considerably larger, as was also the quantity of sulphuric and phosphoric acids.—*Wratsh.*

SOME ACCIDENTS IN OVARIOTOMY.

1. Susanne K., thirty-three years of age, entered the Dorpat hospital with a large abdominal tumor. Some months after her third confinement, she noticed a small movable tumor above the symphysis pubis, which increased slowly. After four years she became pregnant again, and the tumor was now situated on the right side, just below the ribs, and sometimes caused great difficulty in respiration. The woman was well nourished, three months pregnant, and the round tumor was movable, and extended from the umbilicus to the ribs. The abdominal walls being separated by incision, a simple cyst was punctured and the long pedicle secured. Deep sutures were passed through the abdominal walls and the peritoneum. Eight days after the operation, the sutures were removed, and the wound healed per primam. It was covered with adhesive plaster and cotton. On the next day the nurse noticed the cotton to be wet, and the patient complained of pain. The patient was immediately given opium and then etherized. The wound was now seen to have opened in its entire length, and the intestines and a large part of the omentum protruded through the opening. A fibrinous exudation covered the intestines, very difficult to remove. The margins of the wound looked entirely healthy, and as fresh as after the operation. Other sutures were introduced, and left for two weeks. The patient recovered, and the pregnancy was not interrupted.

2. Anna M., twenty-six years of age, noticed a tumor in the abdomen about a year ago. Shortly afterward she became pregnant, and the confinement was normal. Abdominal palpation showed a tumor extending from the symphysis pubis to the umbilicus, and composed of nodes as large as an apple. The vaginal portion was situated on the left side, and nodular tumors were felt throughout the vagina. The uterus was normal and movable, the tumor being moved without difficulty. The diagnosis was multilobular cyst of the left ovary, probably carcinomatous and adherent. In the operation following, the tumor was found to be adherent to the omentum, and intimately connected with the colon ascendens. The pedicle was first secured, and all adhesions to the omentum separated. Then the adherent piece of the mesocolon was secured by two spring-pincettes, and the intestine excised. Large sponges prevented the entrance of blood into the peritoneal cavity. The tumor was now removed, and the mesocolon united by twenty-three sutures. The tumor was a dermoid cyst, containining long hairs and the nodules of an alveolar carcinoma. The woman recovered, but later another tumor appeared on the right side.—*St. Petersburg Med. Wochenschr.*

EXTIRPATION OF A MYO-FIBROMA OF THE UTERUS PER VAGINAM.

Dr. Mikulicz, of Krakow, reports the following case: The patient, a woman 29 years of age, suffered from hæmorrhages for a year and a half. Intense pains came on afterward, and the patient noticed a tumor over the symphysis. She was of medium size, and very anæmic. By palpation of the abdominal walls a tumor was felt as large as a child's head; per vaginam a tumor of the size of a fist was found situated in the posterior wall of the uterus. The cavity of the uterus reached as high as the navel; and a part of the tumor was imbedded in the cervix. The diagnosis was intramural myoma of the posterior wall of the whole uterus. The operation was performed as follows: The perinæum was dissected down to the sphincter ani, and the greater part of the posterior vaginal wall down to the rectum. The lower part of the tumor was then seized and by two lateral cuts its capsule was entered. The latter was enucleated in the usual way by entering the capsule with blunt instruments and tearing the tumor down with the forceps. The upper part offered considerable resistance, and it was only possible to extirpate the growth in fragments. Then the whole hand entered the cavity of the tumor and the rest was enucleated by the fingers. But there was another subserous tumor intimately connected with the large intramural growth. The uterus was therefore inverted and the rest of the intramural and the whole subserous tumor removed by scissors. The peritoneal wound was sutured by carbolized catgut, which was easily done on the inverted uterus. The sutured part was then washed with a 3 per cent. carbolic solution, and iodoform applied to the wound, the fundus uteri re-opened, and three drainage-tubes inserted. The vaginal wall and the perinoum were then sutured with catgut and a compresses applied to the abdomen. There were no febrile nor septic results. After three weeks the patient left the bed, and, in 30 days, the hospital.—*Wiener Med. Wochenschrift.*

HÆMATOMA OF THE PANCREAS.

The patient, 40 years of age, had acquired an acute gastritis, by taking a very hearty meal. A few months after, he noticed a tumor growing in the region of the stomach. Examination showed, between the umbilicus and processus xiphoides, a tumor which was fluctuating and of the size of two fists. The course of the disease did not indicate a sarcoma or an abscess, but a cystic tumor of the pancreas was most probable. The abdominal walls having been dissected the transverse colon was separated from the stomach and a solid cyst laid bare. To the anterior surface of this cyst the parietal peritoneum was affixed by sutures, and in this procedure a dark ink-colored fluid flowed through a deep suture; 1900 c.c. of a similar fluid were removed, and the walls of the cyst split open. The internal surface was smooth; near the colon were some ragged masses. The cavity was washed out and an antiseptic treatment followed. After the first few days, a bloody serum was discharged and some black masses expelled, but the course of the disease was afebrile. The discharged fluid was recognized as altered blood, showing hemine by chemical analysis, and by the spectrum. After a few days an eczema appeared around the wound such as is seen in fistula of the stomach; and the discharged fluid showed all the characteristics of the pancreatic fluid (digesting albumen, forming leucine and tyrosine, and changing amylum into sugar). The patient has at the present time a fistula 3 cm. long and 4 mm. broad, which is probably a receptaculum for the pancreatic fluid, while the ductus is probably closed. The tumor was a hæmatoma of the pancreas.—*Weiner Med. Wochenstchrift.*

UNILATERAL ALBUMINAR RETINITIS.

The patient, a soldier, forty-three years of age, entered the hospital with symptoms of profound cachexia. General anasarca, with difficult respiration, and a large amount of albumen in the urine, indicated the presence of kidney disease. On further examination, it was found that there was but one kidney, which was attacked with parenchymatous nephritis. The patient believed his trouble to have originated from a bilateral epididymitis, and there was in fact an induration of both testicles, with chronic hydro-vaginalitis of the right side. The prostate was also indurated in some parts, and generally hypertrophied. To insure the correctness of this diagnosis, the eyes of the patient were examined by the ophthalmoscope. The right eye was perfectly normal, the cornea and lens being clearly transparent, and the choroidea and retina in a healthy condition. The left eye, however, showed all the lesions peculiar to albuminar retinitis. There were two oval plaques of whitish-yellow color situated behind the vessels in the external layers of the retina. The retinal vessels were seen, thus contrasted, even more clearly than usual. The seat of the lesion was doubtless in the granular layer, and of a degenerative character. Another spot, of a blackish-yellow contour, was found on the superior part of the papilla, in the angle formed by the bifurcation of the two ascendant branches of the superior artery, and this spot was produced by fatty degeneration of the external elements of this part of the retina. Visual troubles were at first wanting, but later there were aggravated symptoms, confined to the left side. In the autopsy, the right kidney was found wanting, and the right eye was perfectly normal, while the left kidney was attacked by parenchymatous nephritis, and the left eye by albuminar retinitis.—*Recueil d' Ophthalmologie.*

HEREDITARY SYPHILIS AND RACHITIS.

The lesions of hereditary syphilis and of rachitis are of the same character, the latter being more developed. In hereditary syphilis there is either cutaneous or mucous ulceration, or there are visceral lesions or desquamative syphilis of the tongue. It is always necessary in cases of rachitis to look for cicatrices and defective second dentition. In rachitis there are three principal types of osseous lesions: (1) hard osteophytes; (2) gelatiniform atrophy; (3) spongeous tissue or true rachitis. But all cases are not the same. Sometimes rachitis begins in a child of three years, without any other lesions. In the first type the bones are normal except the ostesphytes which are of considerable size, and situated mostly on the lower half of the humerus and on the internal aspect of the tibia. In the region of the apophysis, there are found one or two spots a millimeter in diameter of a friable chondro-calcareous substance, which is a calcareous infiltration of the cartilage tissue. These lesions are observed in the fœtus and in children up to the sixth week of age. Later there is found gelatiniform atrophy, certain parts of the bone being filled with a yellowish red tissue. The bones of the cranium are often perforated by this mass. On the extremities of the long bones there is sometimes true juxta-epiphyseal luxation, a syphilitic pseudo-paralysis. In the third stage the extremities of the long bones are entirely changed, the tissue being spongiform. Of 100 cases of rachitis, there are found syphilitic lesions in 90, and the other ten have osseous changes only.—*Le Praticien.*

### CEREBRO-SPINAL SYPHILIS AND ITS SPECIFIC TREATMENT.

In modern neuro-pathology a knowledge of no diseases has made more rapid progress than that concerning cerebro-spinal syphilis. A good many well observed cases have been reported in the last twenty years. Bouchat found in three months four developed cases. The first patient had all the symptoms of common hemiplegia, resulting from an attack of apoplexy without any nervous troubles and without any other lesion. After taking mercury and iodide of potassium for a few days, the mobility of the limbs returned, and the patient, after a short time, was cured. This patient had, in the first instance, been declared incurable. Another paralytic person, a child one year old, with delirium agitans and epileptiform attacks, accompanied by hemiplegia of the left side of the face and of the extremities, showed suddenly a paralysis of the third pair on the right side ; and then the syphilitic origin of the malady was ascertained. This coincidence, namely, the ocular paralysis on the right side and hemiplegia on the left, was of course an indication of bilateral lesions on the base of the brain. The third patient was attacked with epileptiform phenomena without loss of consciousness, and no cephalic pain. This epilepsy was circumscribed by localization as affecting certain muscles of the face. The patient had frequent vomiting, amblyopia at intervals, and partial loss of memory. By taking iodide of potassium and mercury, the attacks diminished, the vomiting ceased, the voice returned and the memory was restored.—*L' Union Médicale.*

THE TREATMENT OF SIMPLE ULCER OF THE STOMACH.

The good results of treatment of simple ulcer of the stomach with milk diet are well known, but there are persons who cannot digest milk, and some are affected by it so that they take a cup of milk to regulate their bowels. In these persons it will be utterly impossible to give a regular lacteal regimen in the usual way. But the introduction into the stomach by œsophageal tubes changes certain conditions entirely. With the œsophageal tube several quarts of milk have been introduced into the stomach, and it has been retained without trouble. The œsophageal tube suppresses the repeated vomiting, and this artificial alimentation will be tolerated for a long time. The same patient who will begin to vomit when taking the glass of milk into his hand will introduce the œsophageal tube into his stomach and pump in a pint of milk without trouble. To avoid a possible rupture of the ulcer by enforced dilatation of the stomach, it is advisable to introduce not more than a pint at once. The convalescent patient can take from 5 to 6 pints of milk a day. But the introduction of the tube, six times a day would be imprudent, and it is, therefore, advisable to introduce the milk in a concentrated state. Recently the cream of the milk has been reduced to a powder, 120 grm. of which are equal to a pint of milk. This powder can be dissolved in a small quantity of water and introduced into the stomach *ad libitum.*—*L' Union Médicale.*

RETENTION OF A FŒTAL HEAD IN THE UTERUS FOR FORTY DAYS.

This patient presented herself apparently in full health, and there was nothing noticed about her save an insupportable stench. The pulse ranged from 72 to 78; the tongue was clear; the temperature normal; the appetite good and digestion perfect. She said that she was delivered of a child, 40 days ago, and that her physician was not able to extract the head as it followed the body, and so cut it off and left her. Examination detected a tumor over the symphysis pubis of the size of a child's head, and the walls of the anteverted uterus gave a crackling sound. Injections into the vagina had been made, and ergot administered without any effect. The cervix was in a normal condition, *i. e.*, contracted to the normal size, while the walls of the body of the uterus containing the fœtal head were enlarged, and gave the crackling sound. During all the time a foul smelling fluid came from the uterus. The cervix was dilated with compressed sponges until the application of instruments was possible. Then a polypus forceps was introduced, and the smaller bones removed. The large bones had to be curved into each other and an incision into the cervix had to be made so as to make extraction possible. The patient never had fever and the temperature was normal through and after the operation. The uterus contracted easily, and a few injections removed the remaining fetid fluid.—*Med. Chir. Centralblatt.*

### Hernia Obturatoria Incarcerata, with Operation and Recovery.

Incarcerated hernia obturatoria is not frequently observed, and the operations for its relief have not been very successful. The following case will, therefore, interest the surgeon. The patient, a lady, 65 years of age, complained of colicky pains and retention of feces with flatus for three days. There was also vomiting and pain in the left lower extremity. The abdomen was tympanitic and the cavity between the adductor longus muscle and the rectus femoris was not as deep as usual. Reposition was not successful. In the region of the foramen ovale there was detected on the next day a small, hard body, and all the other symptoms continuing, hernia obturatoria was diagnosticated. An incision was made from the lower margin of the os pubis on the adductor longus, the latter separated, and the pectineus elevated by blunt instruments. Severe hæmorrhage set in. The sac was opened with blunt instruments, but there were some parts on the margin of the sac which were very hard, and whose separation was found to be very difficult. The intestine was reduced and the wound covered with iodoform and cotton. The wound healed but slowly, the tissue being of unhealthy appearance. The woman recovered entirely.—*Aerztl. Intellig.-Blatt.*

SPONTANEOUS SEPTICÆMIA ORIGINATING FROM EXTRA-PUERPERAL SALPINGO-PERITONITIS.

The patient, 33 years of age, had a chill the day before she entered hospital. Ten years ago she was delivered of a child; and she contracted afterward, by hard work, a prolapsus uteri. She has now some red spots in the face, and she suffers from continued vomiting. There is high fever, pulse 132, exanthema on the chest, and the sphincter and os vesicæ are uncontrollable. The following day the exanthema is of a violet color and more diffuse. There was some cough. The next day some pains in the abdomen; lungs affected; after three days she died. The autopsy showed all the symptoms of septicæmia, suppurative peritonitis, resulting from suppurative salpingitis, bilateral pleuro-pneumonia. nephritis parenchymatosa, and œdema pulmonum. The suppurative peritonitis which induced the septicæmia with resulting death was here caused by the suppurative salpingitis which latter was probably produced by traumatic influence. A puerperal origin is here excluded, the person having not been pregnant for the last ten years. But the rapid course of the disease is similar to that of puerperal peritonitis, and the septic infection had been of an intense character.—*Aerztl. Intellegenzbl.*

MOLLUSCUM FIBROSUM.

The patient, a mariner twenty-four years, has never had syphilis, and was of an exceedingly healthy family. In 1879, while starting on a tour in the West Indies, he noticed some nodes on his hands, some of which grew rapidly. Then they remained as they are now. These nodes are dispersed over the whole body, and while they are more confluent on its anterior surface they are more discrete on the back, and there are but a few on the forearms and legs. On the thorax, they form a group of nodules resembling plums, which are cut through in the middle and giving the surface an undulating aspect. Their consistency differs according to their volume. The large ones are pendulous, and they seemingly contain fluid, the small ones being hard. These tumors are all sessile. The skin retains its color, except at the level of the largest tumors, where it is violet and glabrous. They are composed of fibers of connective tissue.—*Gazette Médicale de Nantes.*

PROLAPSUS OF GASTRIC MUCOUS MEMBRANE THROUGH THE UMBILICUS.

A boy, thirteen years of age, had a reddish tumor of the umbilicus, covered with mucous membrane. The tumor was fastened to the umbilicus by a small pedicle, and it secreted a turbid, sour reacting juice. This secretion was increased by contact, and after meals, when the tumor was larger and more highly colored. Fibrine was dissolved by the sour reacting fluid. The extirpated tumor was microscopically examined, and proved to consist externally of the gastric mucous membrane, and internally of the muscular and serous layer of the stomach. The tumor had been noticed after detachment of the umbilical cord from the child. The prolapsus of the pyloric portion had probably been provoked by a dislocation of the pylorus near the umbilical cord, and the midwife had probably caused the total prolapsus by cutting the umbilical cord too short.—*Deutsche Zeitschr. f. Chirurg.*

PAIN IN RIGHT ILIAC REGION.

A servant girl, 19 years of age, complained of pain in the side and in the region of the right anterior superior spinous process of the ilium, as far as the knee. There was no fever, but slight tenderness on pressure. Warm baths increased the pain, and the faradic current was applied without any result. Then a large magnet was attached and the pain ceased immediately. The same experiment was always followed by the same results, but when the magnet was heated no relief was attained. Then ice was placed on the the painful region, with the same good results, and the author thinks that only the reduction of the temperature on the applied part gave the relief. All the other remedies failing, at last a single hypodermic injection of morphine cured the patient.—*Med. Chir. Centralblatt.*

ENCHONDROMA OF THE PAROTID.

Nusbaum calls attention to the following points: After extirpation of a sarcomatous testicle the parotid is mostly suddenly attacked, and, *vice versa*, after expiration of the parotid, sarcoma of the testicle will appear. In operating on the parotid the pes anserinus ought not to be touched. It is rather advisable to leave a small part of the enchondroma in the tissue, and to operate rather with the fingers than with scissors. The lesion of the facialis is a deleterious accident, resulting in incurable paralysis, with the mouth and the eye of the same side half opened and saliva flowing continuously from the mouth. His Royal Highness Duke of Bavaria, Dr. Carl Theodor, is a very able operator in extirpation of tumors of the parotid.—*Aerztl. Intelligenzbl.*

RETENTION OF PLACENTA; INJECTIONS, FOLLOWED BY DEATH.

An anæmic woman, thirty years of age, entered the hospital under the following circumstances: Two weeks ago, two children were born, and two placentæ expelled. But two days afterward a third child was expelled, but the placenta remained. A fœtid fluid was discharged after that time. There was tumefaction of the whole genital tract, and the placenta was enclosed by the contracted cervix The placenta was seized by forceps, but on account of severe hæmorrhage, a tampon was applied. Injections into the vagina and uterus were made. Suddenly she had a high fever, and died three days after. The autopsy showed peritonitis, with a fluid in the abdominal cavity which had entered through the fallopian tube.—*Le Praticien.*

PROLAPSUS RECTI CURED BY A NEW OPERATION.

The general treatment of prolapsus recti is excision of the protruding part and suturing the rectal mucous membrane to the sphincter. Dr. D'Antona has performed with success the following operation on a woman: Seizing the prolapse with four Billroth's pincettes, and forming thus two cylinders of the rectal canal, he introduced one catgut suture into both cylinders and then into the margin of the anus. Another suture is passed through the middle part of one cylinder, carried through the Douglas sac, and the perirectal tissue, returning to the other cylinder. The patient is discharged, cured in 15 days.—*Il Morgagni.*

PAPILLOMA OF THE BLADDER.

The patient, 43 years of age, has suffered for six months from hæmorrhages through the urethra, with evacuation of tissue corpuscules. By examination per rectum, a tumor on the left side of the bladder was found. For three days after examination there was severe hæmorrhage from the bladder. Entering the bladder through the opened perinæum the introduced finger felt a pedunculated tumor which was detached by torsions with the forceps. The tumor was of the size of a walnut, and it proved to be of benignant character. The patient was discharged entirely cured.—*Archives Générales.*

ECHINOCOCCUS IN THE RIGHT PLEURAL CAVITY.

A boy, 12 years old, was taken sick with symptoms of pleuritis. The right side of the thorax was found to be dilated, and the intercostal spaces enlarged. Respiration was retarded, but there was little albumen in the urine. He had suffered from malaria for a long time. At the autopsy a thick serous fluid was found in the pleural cavity; and in the region of the fifth intercostal space there was an echinococcus cyst containing a clear fluid and many echinococci. The right lung was atrophied and the bronchial tube of the same side reduced to a mere filament.—*Lo Sperimentale.*

ERYTHEMA MULTIFORME AND SYPHILIS.

Prof. Neuman publishes a series of cases in which erythema multiforme and nodosum appeared in syphilitic persons right after or during the treatment of syphilis. These cases seemed to prove that the syphilitic diathesis changed the blood and lymph vessels of these persons so as to provoke erythema. The cases were observed in the same ward and under similar circumstances.—*Med. Chir. Centr. Blatt.*

TIC DE SALAAM.

Tic de salaam, or eclampsia mutans,is of rare occurrence. The attack begins with a certain restlesness, the eyes are then widely opened and fixed on certain objects. Then the head is suddenly flexed to the thorax, and the upper body bent forward, while the arms are thrown forward. These movements are rapidly executed, and they are repeated from 10 to 30 times. Consciousness is perfect. The disease has hitherto proved incurable.—*Revue Médicale.*

### DOUBLE PLACENTA.

After the birth of the child a large placenta with the cord attached was delivered, and the physician naturally thought everything all right. But, half an hour afterward, labor-pains began again, and there was expelled another placenta about half as large as the former, and without a cord. The blood vessels in the cord, as well as in both placentæ, were exceedingly small. Both specimens were presented at a meeting of the Academy at Paris. —*Archives Générales.*

### MALARIA IN FLOWER POTS.

Dr. Eichwald, of St. Petersburg, reports the case of a lady who lived constantly ir a room filled with flowers in pots, and who thus acquired an intermittent fever with symptoms of true malaria. It is not the smell of the flowers which produces sickness, as generally believed, but the dampness emanating from the earth in the pots. In this case the lady lived in a region absolutely free from malaria and other fevers.—*Med. Chir. Centl. Blatt.*

### ON TRANSPLANTATION OF MUSCLES.

After extirpation of a large fibro-sarcoma from the superior part of the biceps brachii muscle a piece of the biceps femoris of a dog was implanted. Three months afterward, the electrical current showed normal behavior of the muscle. The implanted muscle proved to be as irritable as the original, and there was a visible contraction of its entire muscle when the elbow-joint was flexed—*Petersburg, Med. Wochenschr.*

